# Machine Learning and Artificial Intelligence in Clinical Medicine—Trends, Impact, and Future Directions

**DOI:** 10.3390/jcm14228137

**Published:** 2025-11-17

**Authors:** Emmanuel Andrès, Carlos Escobar, Kent Doi

**Affiliations:** 1 Internal Medicine Department, University Hospital Strasbourg, 67000 Strasbourg, France; 2 Cardiology Department, University Hospital La Paz-Carlos III, 28046 Madrid, Spain; 3 Department of Emergency and Critical Care Medicine, University of Tokyo, Tokyo 113-8654, Japan

Over the past decade, the integration of machine learning (ML) and artificial intelligence (AI) into clinical medicine has accelerated dramatically, reshaping the ways in which clinicians collect, analyze, and interpret health data. Between 2021 and 2025, more than 53,000 publications have addressed AI, deep learning (DL), and ML in medical contexts across nearly all specialties—from cardiology and neurology to oncology and infectious diseases. Among these, 659 papers have achieved high citation counts, and 28 have been designated as “hot papers”, underscoring the growing influence and impact of AI in modern healthcare.

In response to this rapidly evolving landscape, the Machine Learning and Artificial Intelligence in Clinical Medicine section of *Journal of Clinical Medicine* was established to provide a rigorously peer-reviewed, multidisciplinary platform for advancing the responsible and effective use of AI in clinical practice. This section brings together clinicians, data scientists, and engineers to foster collaboration that enhances patient outcomes and deepens medical knowledge. Its overarching mission is to translate cutting-edge computational research into tangible clinical benefits, ensuring that technological innovation remains aligned with patient safety, ethical integrity, and scientific rigor.

In keeping with this vision, the section welcomes contributions spanning a wide spectrum of AI-driven clinical research. Topics include the development and validation of AI and ML algorithms for disease diagnosis, prognosis, and treatment optimization; predictive and personalized medicine through risk assessment and patient-specific modeling; medical imaging and signal processing applications using deep learning for image segmentation and classification; clinical decision support systems that integrate AI tools into real-world workflows; and digital health innovations, such as AI-powered wearables and remote monitoring systems. Collectively, these domains reflect the section’s commitment to bridging technical advancement with everyday clinical relevance.

Recent trends in the literature highlight the transformative scope of these efforts. Highly cited works such as “AI in Health and Medicine” (2022, 1327 citations, data source: Web of Science) and “Comparing Physician and Artificial Intelligence Chatbot Responses to Patient Questions” (2023, 1386 citations, data source: Web of Science) demonstrate AI’s potential in diagnostics, patient communication, and decision support. Conversely, critical analyses like “Assessment of GPT-4 as a Clinical Chatbot” (2023, 962 citations, data source: Web of Science) remind us that progress must be balanced by rigorous evaluation of ethical, technical, and safety concerns.

A dominant focus in recent years has been AI in medical imaging, where deep learning approaches are revolutionizing radiology, pathology, and cardiology. Publications such as “Deep Learning-Enabled Medical Computer Vision” (2021, 670 citations, data source: Web of Science) and “Explainable Artificial Intelligence in Deep Learning-Based Medical Image Analysis” (2022, 619 citations, data source: Web of Science) emphasize that interpretability is as important as performance for clinical implementation. The growing field of explainable AI (XAI) exemplifies this principle, as transparency and trustworthiness are increasingly recognized as prerequisites for real-world adoption.

Equally significant has been the standardization of reporting and methodological rigor in AI-driven clinical research. The TRIPOD+AI statement (2024, 653 citations, data source: Web of Science) represents a major step toward improving reproducibility, data transparency, and bias mitigation—key elements for building a robust evidence base for clinical AI.

Among the tens of thousands of studies published from 2021 to 2025, a subset of highly cited papers has defined the current trajectory of AI in medicine. Table 1 highlights the ten most cited articles, reflecting central research directions such as AI-driven medical imaging, clinical prediction models, explainable AI, and AI-mediated patient interaction. Together, these works not only demonstrate the breadth of scientific activity in this field, but also its movement toward practical, ethical, and sustainable clinical integration.

Looking forward, this section aims to continue serving as a bridge between data science and clinical care, emphasizing collaboration, transparency, and innovation. By encouraging submissions that combine methodological excellence with clinical insight, *Journal of Clinical Medicine* seeks to ensure that AI technologies are developed and implemented in ways that empower clinicians, protect patients, and advance evidence-based medicine.

## Editorial Board Members’ Note

As editors of this section, we are deeply committed to fostering a scientific environment that balances innovation with clinical responsibility. The rapid progress of AI in healthcare presents both remarkable opportunities and complex challenges—ethical, technical, and organizational. Our mission is to guide this transformation toward measurable patient benefit, equity, and reliability.

We warmly invite researchers and clinicians to contribute to this evolving dialog by submitting original studies, systematic reviews, and visionary perspectives that advance the responsible integration of AI and ML in clinical medicine.

## Figures and Tables

**Table 1:** Top 10 most highly cited papers on AI and machine learning in clinical medicine (2021–2025). Data current as of October 2025. Submission date: 30 October 2025, data source: Web of Science.

Rank	Publication Title	Year	Citations	DOI
**1**	AI in health and medicine	2022	1327	10.1038/s41591-021-01614-0
**2**	Comparing Physician and Artificial Intelligence Chatbot Responses to Patient Questions Posted to a Public Social Media Forum	2023	1386	10.1001/jamainternmed.2023.1838
**3**	Benefits, Limits, and Risks of GPT-4 as an AI Chatbot for Medicine	2023	962	10.1056/NEJMsr2214184
**4**	Review of Artificial Intelligence Techniques in Imaging Data Acquisition, Segmentation, and Diagnosis for COVID-19	2021	809	10.1109/RBME.2020.2987975
**5**	Deep learning-enabled medical computer vision	2021	670	10.1038/s41746-020-00376-2
**6**	TRIPOD plus AI statement: Updated guidance for reporting clinical prediction models that use regression or machine learning methods	2024	653	10.1136/bmj-2023-078378
**7**	Artificial Intelligence and Machine Learning in Clinical Medicine, 2023	2023	636	10.1056/NEJMra2302038
**8**	Explainable artificial intelligence (XAI) in deep learning-based medical image analysis	2022	619	10.1016/j.media.2022.102470
**9**	Recent advances and clinical applications of deep learning in medical image analysis	2022	576	10.1016/j.media.2022.102444
**10**	A review of medical image data augmentation techniques for deep learning applications	2021	572	10.1111/1754-9485.13261

